# From Germinated Pseudocereals to Functionalized Bread: The Role of the Food Matrix in Protein and Phenolic Bioaccessibility

**DOI:** 10.3390/foods15183280

**Published:** 2026-09-17

**Authors:** Sena Roberts, Funda Elmacıoğlu, Timur Hakan Barak

**Affiliations:** 1 Department of Nutrition and Dietetics, Faculty of Health Sciences, Acibadem Mehmet Ali Aydinlar University, 34752 Istanbul, Türkiye; sena.roberts@acibadem.edu.tr; 2 Department of Nutrition and Dietetics, Graduate School, Istinye University, 34010 Istanbul, Türkiye; 3 Department of Nutrition and Dietetics, Faculty of Health Sciences, Istinye University, 34010 Istanbul, Türkiye; felmacioglu@istinye.edu.tr; 4 Department of Pharmacognosy, Faculty of Pharmacy, Acibadem Mehmet Ali Aydinlar University, 34752 Istanbul, Türkiye

**Keywords:** pseudocereal, germination, food matrix, in vitro protein bioaccessibility, bioactive compound bioaccessibility

## Abstract

Despite the well-documented nutritional benefits of germinated pseudocereals, whether these benefits are preserved following bread incorporation remains unexplored. Bread incorporating germinated quinoa, amaranth, and buckwheat at 15% and 30% substitution was evaluated alongside the germinated materials using rheological, chemical, and in vitro digestion simulation methods. Except for amaranth, all formulations demonstrated lower hardness than the whole wheat control. Overall, quinoa and buckwheat breads yielded acceptable rheological quality. Germinated buckwheat exhibited the highest protein content (33.38 g/100 g DW); however, the protein contribution in incorporated bread remained below theoretically expected values. Germinated quinoa (61.5%) showed the highest in vitro protein bioaccessibility, and protein digestibility decreased with increasing substitution levels. Despite the lowest quantitative protein contribution, buckwheat-incorporated bread exhibited the highest protein digestibility among all formulations. Furthermore, buckwheat showed the highest phenolic content (1988 mg GAE/100 g) and flavonoid bioaccessibility (92.7%); however, these advantages were not reflected in the incorporated bread products. Germination-induced improvements do not necessarily translate into enhanced nutrient bioaccessibility in the final bread product. These findings are consistent with a critical role of the food matrix and suggest that germinated quinoa demonstrates the most consistent overall performance, while buckwheat shows advantages in protein digestibility and flavonoid bioaccessibility.

## 1. Introduction

Cereals represent a major source of energy and nutrients for populations worldwide; however, they present marked nutritional limitations, including an incomplete amino acid profile, restricted bioactive compound content, and the predominance of key nutrients such as minerals in bound, poorly bioavailable forms, with further losses occurring during processing [1,2,3,4,5,6].

Pseudocereals have been consumed for thousands of years and are characterized by favorable agronomic sustainability and a nutritional profile distinct from that of cereals, despite sharing comparable starch content and processing characteristics. Although pseudocereals have been proposed as cereal substitutes, their relatively high antinutritional factor content restricts their direct application in food systems and frequently requires preprocessing, adding cost and resource expenditure. Germination represents a low-cost, easily applicable processing method that effectively minimizes antinutritional factors and improves the nutritional properties of pseudocereals, with an expanding body of literature consistently reporting favorable outcomes [7,8,9,10,11].

Developing functionalized bread products [12] through the incorporation of germinated pseudocereal materials has gained increasing attention as a strategy to enhance the nutritional profile of a widely consumed staple food. Previous studies have reported that a 15–30% substitution level of germinated pseudocereal flours yields improved bioactive compound content and protein quality and acceptable sensory and rheological properties. However, the functionalized character of such products is frequently inferred from the nutritional properties of the germinated raw ingredient rather than from evidence obtained from the final product. Enhanced protein, mineral, and bioactive compound bioaccessibility following germination is among the most consistently reported benefits in the literature; however, most existing studies have assessed bioaccessibility or bioavailability in raw germinated materials rather than in processed, consumer-ready products [8,13,14,15,16]. This gap leaves unanswered the critical question of whether germination-induced improvements are preserved through to the final product. Evaluating bioaccessibility in raw germinated ingredients inherently overlooks the food matrix interactions and processing effects encountered during subsequent preparation. Furthermore, to our knowledge, no study has simultaneously evaluated the transfer of germination-induced improvements in protein and phenolic bioaccessibility from three pseudocereals—quinoa, amaranth, and buckwheat—to a final incorporated bread product under identical processing conditions nor quantified this transfer using a recovery-based framework.

This study therefore aimed to evaluate the bioaccessibility of proteins and bioactive compounds, reported to be enhanced by germination, in the final bread product and assess the role of food matrix interactions and processing conditions in determining these outcomes.

## 2. Materials and Methods

The study materials comprised quinoa, buckwheat, and amaranth seeds, which are classified as pseudocereals in the literature. Bread incorporated with germinated pseudocereal flours at different substitution levels was prepared and analyzed. For comparative analysis, 100% whole wheat bread was used as the control.

### 2.1. Procurement of Materials

Commercially available white quinoa and amaranth were germinated following the procedure described below, whereas buckwheat was sourced in its commercially available pregerminated form. Other ingredients required for bread preparation (whole wheat flour, salt, and yeast) were purchased from local markets following the applied method. Incorporated bread was prepared using whole wheat flour alongside germinated pseudocereal flours at the specified substitution levels.

### 2.2. Procedure of Germination

No standardized germination protocol exists for pseudocereals; germination conditions differ across studies, as different temperature and humidity parameters stimulate distinct enzymatic processes and yield different outcomes [14]. In this study, the germination procedure was based on the principles of Majzoobi et al. [17], mainly aiming to achieve enzymatic activation and enhance bioactive compound and amino acid bioaccessibility. Germination was performed in a Nuve Test Cabinet TK-252 (Nüve Laboratory and Sterilization Technology, Ankara, Türkiye). As the warm and humid conditions required for enzymatic activation simultaneously favor pathogenic bacterial and fungal growth [16], all pseudocereal seeds were surface-sterilized before germination. Sterilization was performed following widely adopted surface decontamination protocols (70% ethanol for 1 min, followed by 1% NaOCl for 10–15 min and rinsing with distilled water) [18]. Sterilized seeds were placed on autoclaved moist filter paper in sterile Petri dishes under a laminar flow cabinet (Deltalab, Barcelona, Spain) and transferred to the test cabinet for germination under species-specific conditions.

Among pseudocereals, quinoa is the most broadly investigated in the context of germination and breadmaking [19,20,21]. White quinoa (Reis Royal White Quinoa, Istanbul, Türkiye) was germinated at 22 °C and 90–95% relative humidity and in darkness for 72 h, as preliminary checks at 48 h indicated insufficient sprout development. From 400 g of raw quinoa, 573 g of germinated quinoa was obtained following imbibition, which yielded 253 g of dried germinated quinoa (moisture content, 53.9%; dry matter, 46.1%).

Germination conditions for amaranth differ across literature [13,22,23]. The germination temperature was set at 26 °C, based on response surface methodology findings reporting this as the optimal temperature for maximizing phenolic content and antioxidant activity in amaranth germination [24]. Considering that amaranth germination conditions are particularly conducive to microbial contamination, particularly fungal growth [25], the abovementioned sterilization procedure was applied. Amaranth was germinated at 26 °C and 90% relative humidity and in darkness for up to 48 h. Germination was monitored at 24 and 48 h; as sprouts reached ideal size by 48 h, the procedure was terminated at this point. Dried germinated amaranth (137.2 g) was generated (moisture content, 48.0%; dry matter, 52.0%).

### 2.3. Procedure of Lyophilization

Following germination, all pseudocereal materials were pre-frozen at −70 °C (Arctiko Uluf P400 MV, Watertown, MA, USA) for 4 days as a pre-lyophilization step. Freeze-drying was subsequently performed for 4 days using a Lyovapor L-200 lyophilizer (BÜCHI Labortechnik AG, Flawil, Switzerland) at a shelf temperature of −60 °C and a chamber pressure of 0.005 mbar. Dry matter content was determined gravimetrically before and after lyophilization. Dried materials were immediately vacuum-packed and stored at −20 °C until use.

### 2.4. Methods

#### 2.4.1. Experiment Planning

Three pseudocereals, including quinoa, amaranth, and buckwheat, were germinated and used for preparing incorporated bread at two substitution levels (15% and 30%), which yielded six incorporated bread formulations in total. To facilitate more comprehensive comparisons across selected analyses, a 100% whole wheat bread was prepared as the control. Bread preparation was based on the AACC 10-10B method [26], incorporating the substitution modifications described by Çetiner et al. [27,28,29].

For bread preparation, 130 g of whole wheat flour was used for the control bread. Incorporated bread contained 20 g of germinated lyophilized flour + 110 g of whole wheat flour at 15% substitution and 40 g of germinated lyophilized flour + 90 g of whole wheat flour at 30% substitution. All formulations contained 1.5% dry yeast and 1.5% salt. Doughs were proofed at 33 °C and 70% relative humidity for 55 min in a proofing chamber (Fimak Bakery and Gastronomy Machines, Istanbul, Türkiye) and subsequently baked with steam at 205 °C for 5 min, followed by 195 °C for 25 min in a professional convection oven (Fimak Bakery and Gastronomy Machines, Istanbul, Türkiye).

#### 2.4.2. Rheological Analyses

*Texture analysis*

Texture analysis of all incorporated bread samples was performed according to AACC methods 74-09 and 74-10 [26] using CTX-CT3 Texture Analyzer (AMETEK Brookfield, Middleboro, MA, USA) with a P1 probe. Measurements were conducted on Days 1, 3, and 5, with three replicates per sample. On Day 5, samples fractured during compression and could not generate valid measurements; consequently, Day 5 data were excluded from the analysis. At the 30% substitution level, fracturing was also observed during the second compression cycle on Day 3. Each measurement comprised two compression cycles at a 1.00 mm/s test speed, which compressed bread to 50% of its original height, from which hardness and springiness values were derived. To characterize initial textural properties, work C1 and deformation parameters were exclusively analyzed for Day 1, as no storage data were available for these parameters. Accordingly, staling behavior was solely assessed on the basis of hardness values across storage days.

*Color analysis*

Crust and crumb color of the control and incorporated bread samples was measured using a High-Quality Colorimeter NR200 (Threenh, Guangzhou, China), following the principles of Nakov et al. [30]. Color was expressed in the CIE L*, a*, b* color space, where L* represents lightness, a* the red–green axis, and b* the yellow–blue axis. Three readings were taken at different points on both the crust and crumb surfaces; mean values were recorded.

#### 2.4.3. Chemical Analyses

*Dry matter determination*

Following germination, dry matter content of germinated materials was determined gravimetrically before and after lyophilization. The dry matter content of bread samples was determined by drying 1.5 cm slices in a single layer at 105 °C for up to 8 h (P-Selecta Digiheat, Barcelona, Spain), with drying terminated upon reaching a constant weight to avoid protein degradation.

*Soluble protein/peptide determination*

Soluble protein content (albumin and globulin fractions) was determined using the Bradford [31] method using Coomassie Brilliant Blue G-250 reagent (Sigma-Aldrich, St. Louis, MO, USA). Albumin was extracted in distilled water (1 g/10 mL) and globulin in 500 mM NaCl in sodium phosphate buffer (1 g/20 mL), both at 4 °C for 4 h with magnetic stirring, followed by centrifugation at 10,000× *g* for 30 min (Allegra 64R, Beckman Coulter, Brea, CA, USA). Absorbance was measured at 595 nm (ThermoScientific Orion AQ-8100, Waltham, MA, USA) against a bovine serum albumin (BSA) standard curve (0–1000 µg/mL). All analyses were performed in triplicate.

*Free amino acid (FAA) determination*

The FAA contents of germinated pseudocereals, incorporated and control bread, and in vitro digestion fractions were determined using the modified o-phthaldialdehyde (OPA) method [32,33]. Samples were extracted in 6% HCl (1 h, room temperature (22 ± 2 °C)), centrifuged at 10,000× *g* for 10 min, and filtered through 0.45 µm syringe filters. OPA reagent was prepared in a 0.1 M borate buffer (pH, 9.5) with 0.1% sodium dodecyl sulfate (SDS) to enhance the solubility of bread protein matrices. L-leucine was used as the standard (0.05–1 mg/mL). Reaction mixture (50 µL sample + 200 µL OPA reagent) was incubated in darkness for 2–3 min; absorbance was measured at 340 nm within 5 min of reaction initiation. All analyses were performed in triplicate.

*Total and soluble sugar content determination*

Total sugar content was determined using the Lane–Eynon titration method (AOAC method 923.09) [34]. Reducing sugar content was measured using the phenol–sulfuric acid method [35].

*Total phenolic (TPC) and flavonoid content (TFC) determination*

The TPC of germinated pseudocereals, incorporated and control bread, and in vitro digestion fractions were determined using the Folin–Ciocalteu colorimetric method [36,37]. For both TPC and TFC analyses, 1 g of dried and ground material was extracted in 20 mL of 80% ethanol (30 min, room temperature (22 ± 2 °C), magnetic stirring) and centrifuged at 10,000× *g* for 10 min (Allegra 64R, Beckman Coulter, Brea, CA, USA), and the supernatant volume was recorded. For TPC, 0.5 mL of the diluted extract was mixed with 2.5 mL of Folin–Ciocalteu reagent and 0.5 mL of 7.5% Na_2_CO_3_ and incubated for 30 min in darkness at room temperature (22 ± 2 °C), and absorbance was measured at 765 nm. Results were expressed as mg GAE/100 g DW. For TFC, 1 mL of the diluted extract was mixed with 1 mL of 2% AlCl_3_ in methanol and incubated in darkness at room temperature, and absorbance was measured at 415 nm against a quercetin standard curve (10–80 µg/mL) [38]. Results are expressed as mg QUE/100 g DW. All measurements were performed using a UV-VIS spectrophotometer (ThermoScientific Orion AQ-8100, Waltham, MA, USA) in triplicate.

#### 2.4.4. In Vitro Phenolic Compound and Protein Bioaccessibility Analyses

In vitro digestion simulation was performed following Barak et al. [39]. Before analysis, approximately 20 g of lyophilized germinated pseudocereal flour was extracted in 50 mL methanol for 72 h and subsequently re-lyophilized (Lyovapor L-200; BÜCHI Labortechnik AG, Flawil, Switzerland), yielding 900 mg (buckwheat), 880 mg (quinoa), and 720 mg (amaranth) of dry material. Samples were stored in 1.5 mL amber glass vials at −20 °C until analysis.

**Figure 1:**
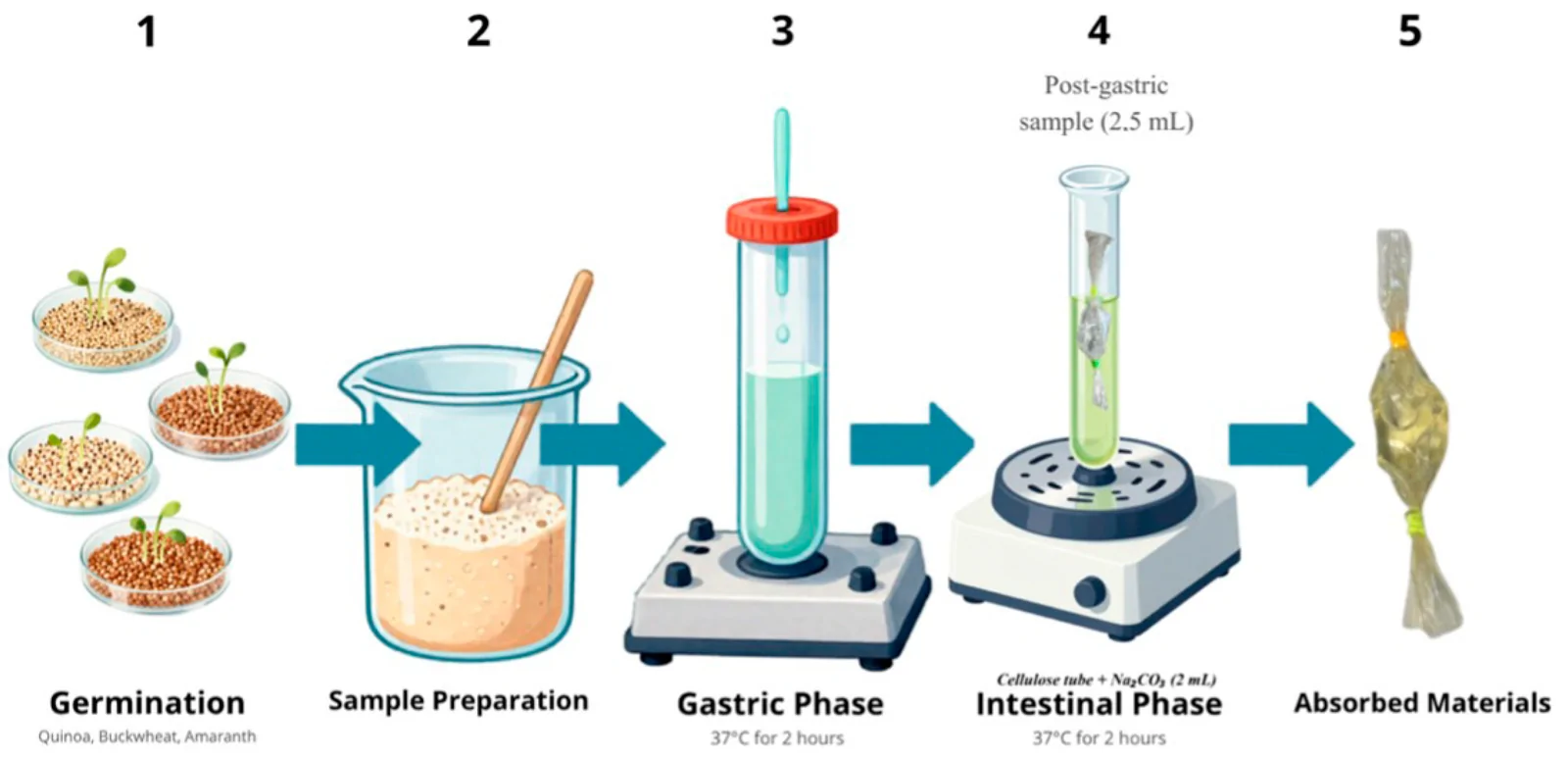
Schematic representation of the in vitro digestion simulation procedure applied to germinated pseudocereal materials.

A 5500 μL sample solution was combined with a certain volume of gastric milieu, including pepsin enzyme and related electrolytes (pH, 2.0). To simulate peristaltic movement, the mixture was incubated in a shaking water bath at 37 °C for 2 h; subsequently, the enzymatic activity was halted by transferring samples to an ice bath. A 2 mL aliquot was collected as the post-gastric (PG) fraction. To simulate intestinal (IN) absorption, a dialysis membrane containing sufficient NaHCO_3_ to neutralize the acidic environment was introduced into the mixture, followed by adding bile acids and pancreatin. Following a further 2 h incubation, the contents of the dialysis membrane were collected as the IN fraction, representing the serum-available, bioaccessible portion. All fractions, including the non-digested (ND) sample, were stored at −20 °C until further analysis. A schematic representation of the in vitro digestion simulation procedure is presented in Figure 1.

Although in vitro digestion simulation was performed on germinated pseudocereal materials to assess protein and bioactive compound bioaccessibility, direct application of this procedure to incorporated bread was not conducted, as the low substitution levels (15% and 30%) would yield negligible detectable contributions. Instead, the impact of incorporation on bioactive compound content was evaluated by calculating the theoretical expected incorporation contribution, the actual incorporation-derived content, and the recovery percentage for each formulation. The theoretical enrichment contribution and recovery percentage for each formulation were calculated relative to the whole bread control, following the approaches described by Fidelis et al. [40] and Nouska et al. [41].

*Protein digestibility degree*

The protein digestibility of the study components was assessed following the method described by Torcello–Gómez et al. [42]. FAA determination was performed using the OPA method as previously described, following the referenced protocol. Post-digestion FAA release, representing the amino acids available for absorption, was expressed as µmol NH_2_ equivalents per mg protein. The degree of hydrolysis (DH%) was calculated according to the OPA method described by Nielsen et al. [43], using BSA as the calibration standard and htot value of 8.1 meqv/g protein. As total amino acid content could not be determined owing to laboratory constraints, it was estimated by multiplying total protein content by a conversion factor of 0.92, as recommended by the Food and Agriculture Organization [44].

### 2.5. Statistical Analysis

Statistical analyses were performed using IBM SPSS Statistics (version 20.0) (IBM Corp., Armonk, NY, USA). Data obtained from germinated pseudocereals, incorporated bread, and the whole wheat bread control were compared within and between groups using one-way ANOVA followed by Tukey’s HSD post hoc test. Differences were considered statistically significant at *p* < 0.05. For hardness data, a two-way mixed ANOVA was applied with bread formulation as the between-subjects factor and storage time as the within-subjects factor, including the formulation × storage time interaction term. Post hoc comparisons were performed using Bonferroni correction.

## 3. Results

### 3.1. Rheological Analysis

#### 3.1.1. Texture

The rheological evaluation centered on the time-dependent staling behavior, hardness, and springiness of the bread samples. The hardness values on Days 1 and 3, alongside the springiness data, are presented in Table 1.

The rheological properties of the incorporated bread varied significantly depending on the grain type and substitution level. The hardness values on Day 1 ranged from 72.59 ± 2.27 N (BB15) to 135.5 ± 3.47 N (AB15), with amaranth-incorporated bread demonstrating significantly higher hardness than quinoa and buckwheat bread at both substitution levels (*p* < 0.001). By Day 3, hardness significantly increased in all formulations (*p* < 0.05), with the exception of AB15, which showed a non-significant increase (*p* = 0.060). Moreover, AB30 unexpectedly decreased in hardness from Day 1 to Day 3 (116.5 ± 0.62 vs. 113.6 ± 0.50 N), which suggests, despite being statistically significant (*p* < 0.001), an atypical staling behavior that can be associated with moisture redistribution or protein–starch interactions specific to high-level amaranth substitution. At the 30% substitution level, quinoa and buckwheat bread were statistically comparable in terms of hardness (*p* = 0.57), whereas amaranth bread remained significantly harder than both (*p* < 0.001). Springiness significantly decreased with increasing substitution levels in quinoa and amaranth bread samples (*p* < 0.001), whereas BB15 and BB30 exhibited no significant difference (*p* = 0.48). Overall, quinoa and buckwheat bread demonstrated acceptable textural profiles across both substitution levels, which supports their potential for functionalized bread development.

#### 3.1.2. Color Analysis

The crust and crumb color parameters (L*, a*, and b*) differed significantly among bread formulations. The incorporated bread samples demonstrated lower L* values than the control bread, indicating a darkening of both the crust and crumbs with pseudocereal incorporation. This darkening tendency was the most pronounced in quinoa bread, wherein L* progressively decreased with increasing substitution levels (from 56.67 at 15% to 49.80 at 30%). Amaranth-incorporated bread showed the highest a* values in the crust (25.38 ± 2.93 at 15%), which reflected a distinctly reddish appearance, alongside the highest b* values across all groups, which indicates a more yellow-toned crumb and crust compared to the other formulations. The color information of the crumb and crusts of bread is presented in Table 2.

### 3.2. Chemical Analyses

The results of the chemical analyses, including those of dry matter, total sugar, reducing sugar, protein, free amino acids, total phenolic compound, and total flavonoid content, are presented in Table 3.

#### 3.2.1. Dry Matter Determination

The dry matter contents of the germinated pseudocereals were 46.1%, 47.3%, and 48.0% for quinoa, buckwheat, and amaranth, respectively. For the incorporated and control bread, dry matter contents were: QB15, 59.2%; QB30, 59.1%; BB15, 61.5%; BB30, 63.5%; AB15, 63.3%; and AB30, 66.5%.

#### 3.2.2. Protein and FAA Determination

Germinated buckwheat (33.38 g/100 g DW) exhibited the highest protein content of the germinated pseudocereals, followed by germinated amaranth (30.34 g/100 g DW) and germinated quinoa (21.25 g/100 g DW). The protein content of the incorporated bread varied according to grain type and substitution level. Quinoa-incorporated bread demonstrated the most marked increase with increasing substitution levels, increasing from 12.10 ± 0.20 g/100 g DW at 15% to 20.82 ± 1.10 g/100 g DW at 30% (72.1% increase). Buckwheat-incorporated bread contained 8.85 ± 0.10 and 13.75 ± 1.30 g/100 g DW at the 15% and 30% substitution levels, respectively (55.4% increase), remaining lower than the other formulations despite the relatively high protein content of the germinated ingredient. Amaranth-incorporated bread showed the highest absolute protein values among the incorporated bread samples (20.95 ± 0.10 and 23.86 ± 0.40 g/100 g DW at 15% and 30%, respectively); however, the increase with substitution level was comparatively modest (13.9%).

**Figure 2:**
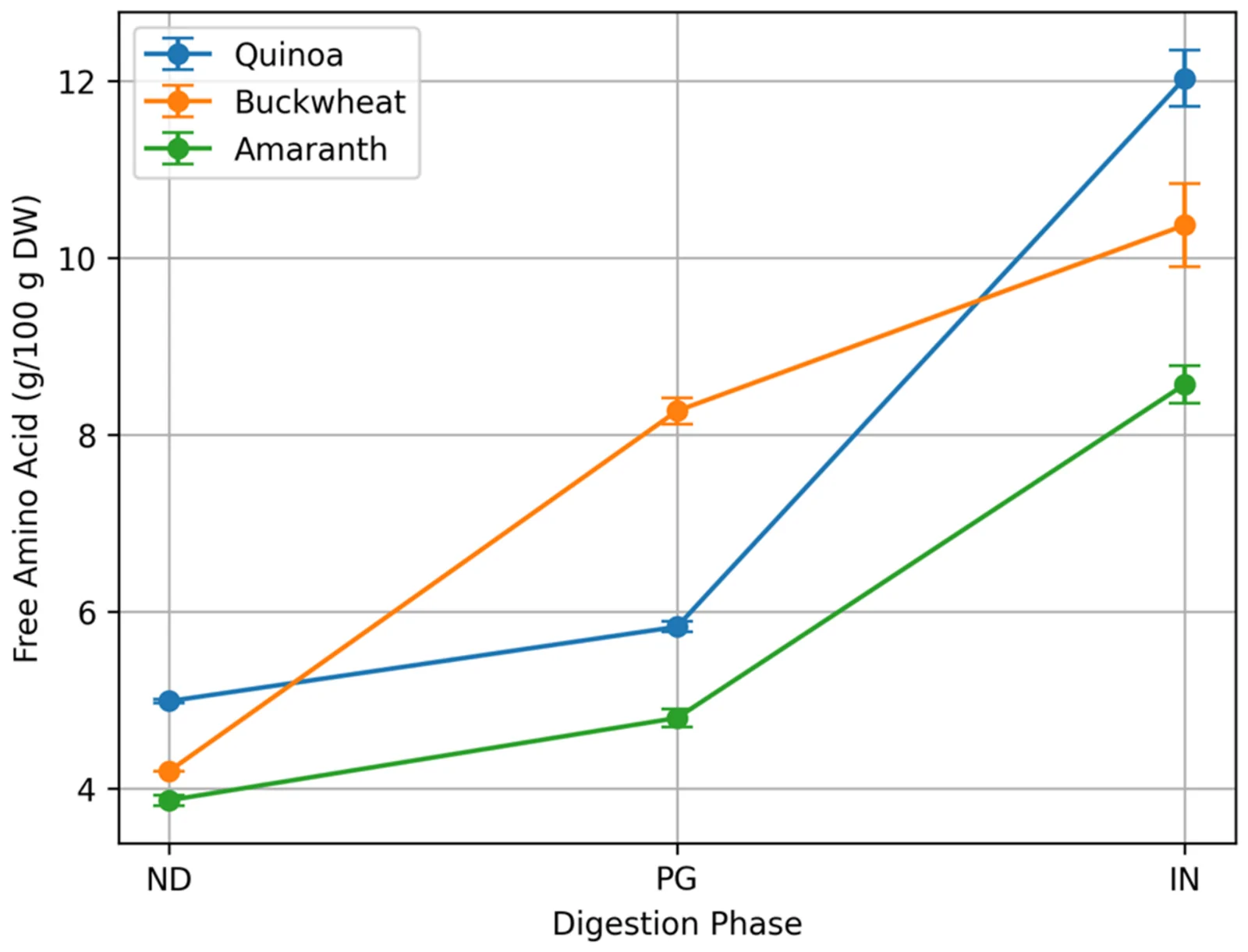
Free amino acid release during in vitro digestion of germinated pseudocereals. DW: dry weight; ND: non-digested; PG: post-gastric; IN: intestinal (serum-available fraction).

Germinated quinoa (4.99 ± 0.10 g/100 g DW) exhibited the highest FAA content of the germinated pseudocereals, followed by germinated buckwheat (4.20 ± 0.00) and germinated amaranth (3.87 ± 0.05). The FAA content of the incorporated bread only marginally increased with the 15% and 30% substitution levels across all grain types: quinoa bread, 7.50 ± 0.70–8.03 ± 0.98; buckwheat bread, 7.13 ± 0.40–7.30 ± 0.10; and amaranth bread, 6.65 ± 1.05–7.53 ± 0.40. In vitro digestion of germinated pseudocereals revealed a consistent and statistically significant increase in FAA release from the ND to the IN phases across all samples (one-way ANOVA, *p* < 0.001); this supports a biologically relevant trend in protein hydrolysis and amino acid liberation during digestion. Of note, this comparison was based on three samples; interpretations should be made accordingly. The free amino acid release across digestion phases is illustrated in Figure 2.

Germinated quinoa (61.5%) exhibited the highest protein bioaccessibility (BAc%) of the germinated pseudocereals, followed by buckwheat (33.7%) and amaranth (30.7%), with all digestion phase differences showing statistical significance (*p* < 0.001; Table S1).

As protein content alone does not reflect the degree of protein digestibility in the final product, the amount of free amino groups released per mg protein was further determined for both the germinated pseudocereals and the incorporated bread. These data were used for estimating the degree of protein digestibility in the bread samples. The estimated protein digestibility values are presented in Table 4.

#### 3.2.3. TPC and TFC

Germinated buckwheat (1988 ± 69 mg GAE/100 g DW) exhibited the highest TPC of the germinated pseudocereals, followed by germinated quinoa (1030 ± 17 mg GAE/100 g DW) and germinated amaranth (717 ± 49 mg GAE/100 g DW). The whole wheat bread control had a TPC of 1106 ± 90 mg GAE/100 g DW. In incorporated bread, TPC increased with substitution across all grain types; however, it remained below the control in most formulations. Buckwheat bread (from 613 ± 45 to 963 ± 86 mg GAE/100 g DW; 57.1% increase) demonstrated the most pronounced increase with substitution level, followed by amaranth (from 692 ± 68 to 828 ± 110 mg GAE/100 g DW; 19.7% increase) and quinoa bread (from 904 ± 56 to 1009 ± 56 mg GAE/100 g DW; 11.6% increase).

TFC followed a different pattern: germinated quinoa showed the highest TFC (359 ± 3 mg QE/100 g DW), markedly exceeding germinated buckwheat (97 ± 1 mg QE/100 g DW) and germinated amaranth (22 ± 0 mg QE/100 g DW). The whole wheat bread control contained 223 ± 22 mg QE/100 g DW. In incorporated bread, TFC increased with increasing substitution levels in all formulations, with the greatest relative increases noted in quinoa (from 88 ± 1.15 to 137 ± 0 mg QE/100 g DW; 55.7% increase) and buckwheat bread (from 38 ± 1.5 to 58 ± 10 mg QE/100 g DW; 52.6% increase), whereas amaranth demonstrated a more limited increase (from 34 ± 1.5 to 45 ± 0.5 mg QE/100 g DW; 32.4% increase). All incorporated bread values remained below those of the whole wheat control.

**Figure 3:**
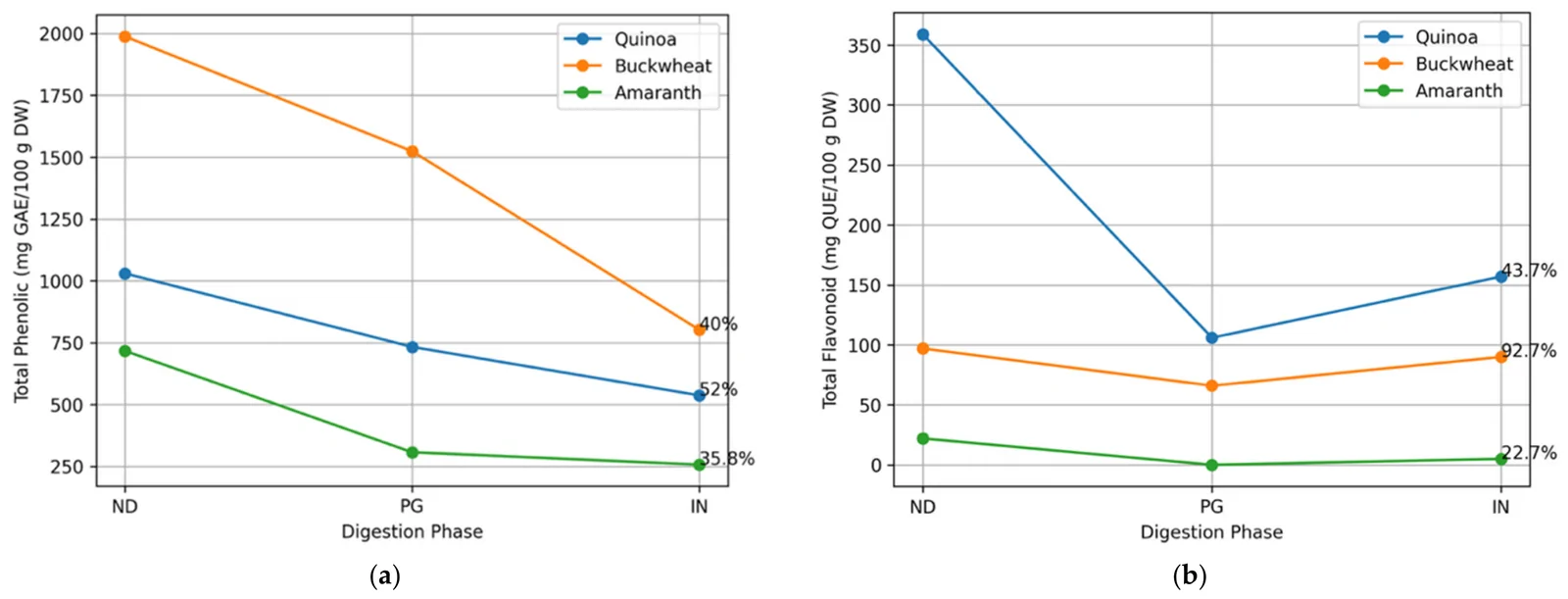
(**a**) Changes in TPC during in vitro digestion phases in germinated pseudocereals; (**b**) changes in TFC during in vitro digestion phases in germinated pseudocereals. DW: dry weight; ND: non-digested; PG: post-gastric; IN: intestinal (serum-available fraction).

During in vitro digestion, TPC substantially decreased across all germinated pseudocereals from the ND to the IN phases. Quinoa (52%) showed the highest bioaccessibility (BAc%), followed by buckwheat (40%) and amaranth (35.8%). TFC revealed a distinct digestion pattern: buckwheat exhibited exceptionally high flavonoid bioaccessibility (92.7%), whereas quinoa (43.7%) and amaranth (22.7%) showed comparatively lower values. Notably, quinoa flavonoids exhibited a pronounced decrease in the PG phase followed by a partial recovery in the IN phase, which suggests phase-dependent stability differences (Figure 3; Table S2).

Theoretical incorporation contributions and recovery rates were calculated relative to the whole wheat bread control to further evaluate the contribution of pseudocereal incorporation to the bioactive compound content of bread (Table 5). For TPC, meaningful recovery was only observed at the 30% substitution level: quinoa bread demonstrated the highest recovery (75.7%), followed by buckwheat (31.4%) and amaranth (24.0%) bread. At the 15% substitution level, no quantifiable incorporation contribution above the control was detected in any formulation. For TFC, recovery was exclusively observed in 30% quinoa bread (19.0%), with other incorporated bread groups showing no detectable contribution. These results suggest that the expected bioactive compound contribution from germinated pseudocereals does not proportionally translate into the bread matrix and that a meaningful phenolic incorporation effect is only achievable at higher substitution levels and predominantly with quinoa.

## 4. Discussion

Incorporating pseudocereals into bread formulations has been widely reported to exert beneficial effects, improving the protein and amino acid profiles and contributing bioactive compounds to the final product. The high antioxidant capacity of pseudocereal components may further help delay oxidative deterioration in bread. Several studies have investigated the thermal properties of pseudocereal ingredient-incorporated bread matrices, with varying results reported across the literature. The characteristic properties and physicochemical contributions of starch and protein fractions generally explain the underlying mechanisms of thermal effects [45,46,47]. Miranda–Ramos and Haros [48] reported increased crumb firmness with increasing substitution levels in bread incorporated with combined quinoa, amaranth, and chia flours, which aligns with the higher hardness values observed in amaranth-incorporated bread in this study. By contrast, quinoa and buckwheat bread exhibited a decreasing hardness trend with increasing substitution levels, which resulted in softening, consistent with findings reported by Akturfan and Yalçın [49]. These findings suggest the grain-dependent effect of substitution level on textural properties, reflecting the distinct interaction mechanisms of quinoa, buckwheat, and amaranth within the gluten matrix. The higher retrogradation tendency and weaker structure-forming capacity of amaranth starch may account for the more pronounced hardness noted in amaranth-incorporated bread [50,51,52,53,54]. Furthermore, the incorporation of non-gluten pseudocereal proteins in the wheat dough system may dilute the gluten network, reducing its viscoelastic continuity and water-binding capacity. At higher incorporation levels, this network disruption becomes more pronounced, as pseudocereal proteins compete with gluten proteins for water and interfere with disulfide bond cross-linking during dough development, which results in a weaker, more rigid crumb structure upon baking [55,56]. The lower hardness values observed in QB30 on Days 1 and 3 compared with the other 30% incorporated bread may be attributed to the smaller starch granule size of quinoa compared to the other pseudocereal starches, which facilitates greater gelatinization and binding capacity within the bread matrix [57].

To evaluate the rheological properties of incorporated bread, color analysis was performed as an additional parameter. Incorporated bread exhibited significantly lower L* values than the whole wheat control, which indicates that darkening proportionally increased with the substitution level. Significantly higher a* and b* values in incorporated bread reflected a redder and yellower-toned appearance than the control, a trend consistent across both crust and crumb measurements and aligning with previously reported findings in the literature [52,53,54,57,58]. Maillard reactions and the contribution of natural pigments inherent to pseudocereal ingredients may explain the darker, redder, and yellower appearance of incorporated bread [59]. In particular, the marked darkening and browning observed in amaranth-incorporated bread crusts may be associated with the relatively high arginine and lysine content of amaranth, as these amino acids are among the most reactive participants in the Maillard reaction [60]. During baking, free amino acids react with reducing sugars—particularly glucose released during germination—through the Maillard reaction cascade, generating melanoidins responsible for the observed color changes. Germination further amplifies this effect by increasing the pool of reactive substrates, intensifying browning in germinated pseudocereal-incorporated bread [61].

Several studies have reported germination-induced elevations in reducing and total sugar contents; however, the reported values vary widely and are frequently based on nonstandardized analytical methods, which renders direct comparison with the present data challenging. Among the three pseudocereals, starch content follows the following order: buckwheat > quinoa > amaranth [62]. A considerable proportion of quinoa and amaranth polysaccharides exist in forms bound to phenolic acids, including ferulic and coumaric acids [63], which partly explains the relatively low monosaccharide release observed in amaranth during germination, owing to both its lower starch content and insufficient bound fraction degradation within the germination period applied. Furthermore, the optimal activity of α-amylase during germination reportedly peaks at approximately 6 days [64]. As the germination periods in this study were shorter, monosaccharide release may not have reached its maximum. Furthermore, the enzymatic breakdown of complex starch granules does not frequently cause increased monosaccharide content, as resistant starch formation may also occur as a competing outcome [65]. During baking, starch gelatinization disrupts the crystalline structure of starch granules, making them more susceptible to enzymatic hydrolysis. However, retrogradation upon cooling can reconstitute resistant starch fractions, particularly in amaranth, where the compact protein–starch matrix may further limit amylase accessibility [14].

Pseudocereals are characterized by a remarkably high protein content relative to that of conventional cereals, with reported values of 11.7–18.4%, 21.6–25.3%, and 13.0–14.5% for amaranth, buckwheat, and quinoa, respectively [66]. Germination further increases protein content and digestibility through enzymatic activation; however, some studies have reported a transient decrease attributed to the enzymatic degradation of antinutritional factors, including saponins [15,67,68]. However, the prevailing consensus supports a net increase in protein content following germination and has been reported to substantially increase protein content in pseudocereals; increases of up to 1.29-fold have been documented in quinoa varieties during germination [69], with amaranth and buckwheat demonstrating comparable increasing trends [15]. The present results align with this trend, with protein contents of 33.38, 30.34, and 21.25 g/100 g DW determined for germinated buckwheat, amaranth, and quinoa, respectively.

Among incorporated bread formulations, germinated buckwheat, despite having the highest protein content among the germinated pseudocereals, contributed the lowest protein content. This finding may be explained by the food matrix characteristics of buckwheat: it contains the highest polyphenol–protein complex ratio among the three pseudocereals, alongside elevated lysine and arginine contents and glucose as the predominant sugar released during germination, conditions that collectively facilitate Maillard reactions during baking. The inherently low thermal stability of buckwheat proteins further supports this explanation [70,71,72,73]. Nevertheless, despite its limited quantitative protein contribution, buckwheat-incorporated bread generated the highest NH_2_ release per mg protein in digestibility calculations, which indicates a constrained protein content but superior digestible protein quality, attributable to the vulnerability of buckwheat proteins to thermal and enzymatic degradation. Specifically, the thermal denaturation of buckwheat proteins leads to the formation of intermolecular disulfide bonds and protein aggregation; while aggregation reduces soluble protein content, the resulting denatured structures are more accessible to digestive proteases; this explains the paradoxical combination of low protein contribution but a high degree of hydrolysis in buckwheat-incorporated bread [74].

Among germinated pseudocereals, quinoa (4.99 g/100 g DW) demonstrated the highest FAA content, followed by buckwheat (4.20 g/100 g DW) and amaranth (3.87 g/100 g DW). The relatively low FAA content compared with total protein aligns with the literature [75,76] and reflects the natural dynamics of germination, during which FAAs are transiently accumulated and actively utilized as metabolic substrates throughout the process.

Incorporated bread exhibited a generally higher FAA content than raw germinated pseudocereals, which potentially reflects protein breakdown during fermentation and baking. However, FAA values remained below theoretically expected levels, potentially attributable to the gluten matrix of whole wheat flour: gluten forms a “continuous complex” with starch granules in the endosperm, serving as a physical barrier against digestive enzymes and processing. This matrix effect may restrict FAA release and detection in incorporated bread samples [77,78]. Pseudocereal incorporation increases protein content and modifies the amino acid profile of bread. Although pseudocereals are characterized by glutamic acid, aspartic acid, arginine, and lysine as dominant amino acids, incorporated bread formulations exhibit a shift toward glutamic acid, proline, leucine, aspartic acid, arginine, serine, and phenylalanine, a pattern reported to differ depending on the incorporation source and processing conditions [53,79]. Among the three pseudocereals, amaranth demonstrated the lowest FAA content, which may be partly explained by the resistance of sulfur-containing protein complexes to enzymatic hydrolysis during germination, as a substantial proportion of amaranth proteins are sulfur-rich [52].

Incorporated bread showed a significantly higher degree of protein digestibility than germinated pseudocereal materials alone; however, it decreased with increasing substitution levels. For example, among incorporated bread, BB15 exhibited the highest digestibility (75.8%), which declined to 50.0% at the 30% substitution level. This inverse association may reflect gluten network disruption by pseudocereal components at higher substitution levels, which causes the formation of tightly aggregated structures resistant to digestive enzymes [80,81]. Additional contributing factors encompass thermal denaturation and disulfide bond formation during baking, which diminish gluten protein digestibility [82] and the potential of food matrix proteins to form complexes with phytochemicals, dietary fiber, phytic acid, lipids, and carbohydrates, further limiting enzymatic access [83]. At higher substitution levels, the increasing concentration of flavonoids and phenolic acids abundant in buckwheat and quinoa may form covalent and non-covalent complexes with both gluten and pseudocereal proteins through oxidative cross-linking reactions. These protein–phenolic complexes exhibit a reduced susceptibility to pepsin and pancreatin hydrolysis, which contributes to the observed decrease in protein digestibility degree [84].

In addition to their protein content, pseudocereals are also valuable sources of bioactive compounds; delivering these compounds into the food matrix represents one of the primary objectives of pseudocereal incorporation in bread. However, the expected health benefits of these bioactive compounds rely on their bioaccessibility and bioavailability, both of which are governed by various factors [85]. As observed for protein, the contribution of TPC to incorporated bread was not proportional to the substitution level. Buckwheat-incorporated bread demonstrated a 57.1% increase in TPC from 15% to 30% substitution; this reflects the high phenolic content of buckwheat. By contrast, quinoa-incorporated bread exhibited only an 11.6% increase over the same range, potentially attributable to the highly conjugated nature of quinoa phenolics, which limits their extractability and contribution to the bread matrix relative to their total content [86].

Pseudocereals are incorporated into bread formulations to enhance nutritional properties and exploit their physicochemical characteristics, with incorporation levels of up to 28% reported to yield increased bioactive compound content, improved protein quality, satisfactory sensory properties, and enhanced digestibility [13,21,71,87,88]. However, the expected incorporation contribution is not frequently fully realized in the final product, which renders the assessment of actual recovery a crucial step in formulation development. Therefore, understanding the conditions under which this contribution is achieved or limited is essential for the development of more effective incorporated bread formulations [40,41]. In this study, no meaningful bioactive compound contribution above the whole wheat control was detected at the 15% substitution level for any formulation. At 30%, recovery varied across grain types, with quinoa bread (75.7%) showing the highest TPC recovery. For TFC, a meaningful recovery value was exclusively obtained in 30% quinoa bread (19%), with no quantifiable contribution in any other formulation.

The values reported in the literature are broadly consistent with the present findings: germinated quinoa reportedly yields gastric and IN phase recoveries of 87.6–116.7% and 89.6–124.5%, respectively, with an absorbable content of 7.4–10.9% and flavonoid recovery of 4.2–12.4% [89]. Bioaccessible phenolic content in germinated buckwheat sprouts reportedly reaches up to 90% [90]. However, direct comparison across studies remains challenging owing to methodological variability; INFOGEST 2.0 has been proposed as a standardized approach for assessing the in vitro bioaccessibility of bioactive compounds [91]. The limited bioactive compound recovery observed in incorporated bread in this study suggests the competing effects of the bread matrix: fermentation and thermal processing can promote phenolic release through protein denaturation while simultaneously causing bioactive compound degradation or enhancing antinutritional factor interactions [92,93,94]. Specifically, the thermal lability of rutin—the predominant flavonoid in buckwheat—renders it susceptible to degradation during baking, with progressive conversion to quercetin observed at temperatures exceeding 80 °C and significant losses reported at typical bread-baking temperatures [95]. The net outcome of these processes relies on the grain type, molecular characteristics of the bioactive compound, and specific processing conditions [94].

Several limitations of the present study should be acknowledged. First, bioactive compound and amino acid analyses were performed on a total content basis rather than profiling individual phenolic compounds or amino acid fractions, which limited the precision of bioaccessibility evaluation and precluded the calculation of the Digestible Indispensable Amino Acid Score (DIAAS); protein quality in enriched breads was therefore assessed via degree of protein digestibility using the OPA method. Second, and most critically, in vitro digestion simulation was not applied directly to the bread samples; the role of the food matrix in modulating bioaccessibility could therefore not be directly demonstrated and should be interpreted with caution. The recovery-based framework applied in this study provides an indirect estimate of incorporation efficiency but does not substitute for experimental bioaccessibility assessment of the final product, and no structural, microstructural, or molecular analyses were conducted to elucidate the underlying matrix-related mechanisms. Accordingly, the conclusions regarding food matrix effects are interpretive rather than mechanistic in nature. Future studies should incorporate direct in vitro digestion of bread matrices alongside structural and molecular analyses, individual phenolic and amino acid profiling, and DIAAS determination to provide a more comprehensive evaluation of the nutritional impact of germinated pseudocereal incorporation.

## 5. Conclusions

This study demonstrates that germination effectively improves the protein and bioactive compound content of quinoa, amaranth, and buckwheat; however, these enhancements do not automatically translate into proportional gains in the final bread product. The findings are consistent with the critical role of the food matrix in protein and phenolic compound bioaccessibility, with the extent of this effect varying by grain type and substitution level. Among the pseudocereals evaluated, quinoa demonstrated the most consistent overall performance across protein bioaccessibility, phenolic recovery, and rheological parameters, which suggests its suitability for functionalized bread development at the substitution levels tested. However, buckwheat exhibited superior protein digestibility and flavonoid bioaccessibility, which highlights the multifactorial nature of pseudocereal selection for bread incorporation and the need to prioritize parameters according to the intended nutritional objective. These findings suggest the critical significance of evaluating bioaccessibility in the final food product rather than in raw or minimally processed ingredients, underlining the need for optimized formulation strategies to maximize the nutritional contribution of germinated pseudocereals in bread matrices.

## Figures and Tables

**Table 1:** Hardness and springiness data of the incorporated bread.

	Hardness (Staling)		Deformation of Hardness (Springiness)
	Day 1	Day 3	Day 1
Whole Wheat Bread	124.2 ± 2.13 ^a^	153.0 ± 2.34 ^a^	24.95 ± 0.01 ^a^
15% Quinoa-Incorporated Bread	84.67 ± 2.13 ^c^	104.39 ± 2.07 ^a^	24.95 ± 0.01 ^a^
30% Quinoa-Incorporated Bread	74.90 ± 3.63 ^d^	90.06 ± 1.19 ^b^	22.45 ± 0.01 ^b^
15% Buckwheat-Incorporated Bread	72.59 ± 2.27 ^e^	89.37 ± 4.95 ^b^	17.49 ± 0.01 ^c^
30% Buckwheat-Incorporated Bread	76.62 ± 3.01 ^d^	103.93 ± 1.05 ^a^	17.51 ± 0.03 ^c^
15% Amaranth-Incorporated Bread	135.5 ± 3.47 ^a^	148.3 ± 7.79 ^a^	24.95 ± 0.01 ^a^
30% Amaranth-Incorporated Bread	116.5 ± 0.62 ^a^	113.6 ± 0.5 ^a^	22.45 ± 0.01 ^b^

Different letters indicate statistically significant differences between the groups (*p* < 0.05).

**Table 2:** Color information of bread samples.

	L*	a*	b*
Crust			
Whole Wheat Bread	57.58 ± 1.53 ^a^	11.93 ± 3.49 ^d^	29.74 ± 3.06 ^c^
15% Quinoa-Incorporated Bread	56.67 ± 0.69 ^a^	13.23 ± 0.39 ^d^	32.95 ± 0.97 ^b^
30% Quinoa-Incorporated Bread	49.80 ± 2.38 ^d^	17.49 ± 3.99 ^c^	34.80 ± 2.56 ^b^
15% Buckwheat-Incorporated Bread	52.83 ± 3.72 ^bc^	10.96 ± 1.79 ^d^	30.09 ± 3.07 ^c^
30% Buckwheat-Incorporated Bread	54.35 ± 4.56 ^ab^	12.30 ± 3.08 ^d^	30.58 ± 6.12 ^c^
15% Amaranth-Incorporated Bread	49.15 ± 2.52 ^d^	25.38 ± 2.93 ^a^	37.62 ± 1.08 ^a^
30% Amaranth-Incorporated Bread	50.24 ± 2.54 ^cd^	21.98 ± 2.09 ^b^	38.49 ± 1.52 ^a^
Crumb			
Whole Wheat Bread	59.13 ± 1.78 ^a^	6.58 ± 0.65 ^cd^	20.85 ± 0.56 ^c^
15% Quinoa-Incorporated Bread	54.97 ± 0.97 ^bc^	9.19 ± 1.57 ^b^	26.44 ± 2.37 ^b^
30% Quinoa-Incorporated Bread	56.93 ± 2.39 ^ab^	6.46 ± 1.05 ^cd^	25.24 ± 0.8 ^b^
15% Buckwheat-Incorporated Bread	57.84 ± 0.25 ^ab^	6.25 ± 0.16 ^d^	19.03 ± 0.84 ^c^
30% Buckwheat-Incorporated Bread	51.83 ± 0.61 ^d^	10.23 ± 3.17 ^ab^	25.22 ± 3.17 ^b^
15% Amaranth-Incorporated Bread	52.62 ± 5.32 ^cd^	7.38 ± 0.73 ^c^	26.45 ± 0.84 ^b^
30% Amaranth-Incorporated Bread	51.02 ± 2.46 ^d^	11.44 ± 4.66 ^a^	32.05 ± 3.06 ^a^

Color parameters (L*, a*, and b*) are analyzed using one-way analysis of variance (ANOVA); differences between the groups are assessed using Tukey’s multiple comparison test. Means within the same column followed by different letters are significantly different.

**Table 3:** Chemical composition of germinated pseudocereals, incorporated bread, and whole wheat bread control.

	Dry Matter (%)	Soluble Sugar (g Glucose eq/100 g DW)	Total Sugar (g/100 g DW)	Soluble Protein (Albumin + Globulin) (g/100 g DW)	Total Protein (g/100 g DW)	Total Amino Acid (0.92) (g/100 g DW)	Free Amino Acid (g/100 g DW)	Total Phenolic Compound (mg GAE/100 g DW)	Total Flavonoid Content (mg QUE/100 g DW)
Germinated Quinoa	46.1	3.8 ± 0.31 ^cd^	7.44	16.6 ± 0.75 ^b^	21.25 ± 0.1 ^c^	19.55	4.99 ± 0.02 ^d^	1030 ± 17 ^b^	359 ± 3 ^a^
Germinated Buckwheat	47.3	1.26 ± 0.32 ^e^	2.68	25.1 ± 0.17 ^a^	33.38 ± 0.1 ^a^	30.71	4.2 ± 0 ^d^	1988 ± 69 ^a^	97 ± 1 ^b^
Germinated Amaranth	48	1.22 ± 0.03 ^e^	1.5	23.7 ± 1.96 ^a^	30.34 ± 0.78 ^b^	27.91	3.87 ± 0.06 ^d^	717 ± 49 ^d^	22 ± 0 ^e^
15% Quinoa-Incorporated Bread	59.2	3.54 ± 0.4 ^d^	2.63	3.20 ± 0.45 ^f^	12.1 ± 0.2 ^e^	11.13	7.5 ± 0.7 ^ab^	904 ± 56 ^c^	88 ± 1.15 ^b^
30% Quinoa-Incorporated Bread	59.1	4.4 ± 0.2 ^c^	2.42	6.73 ± 2 ^d^	20.82 ± 1.1 ^c^	19.15	8.03 ± 0.98 ^a^	1009 ± 56 ^b^	137 ± 0 ^a^
15% Buckwheat-Incorporated Bread	61.6	0.97 ± 0.25 ^e^	2.15	2.33 ± 0.32 ^f^	8.85 ± 0.1 ^f^	8.14	7.13 ± 0.40 ^b^	613 ± 45 ^e^	38 ± 1.5 ^d^
30% Buckwheat-Incorporated Bread	63.5	4.97 ± 0.21 ^c^	2.72	4.47 ± 1.34 ^e^	13.75 ± 1.3 ^e^	12.65	7.3 ± 0.1 ^ab^	963 ± 86 ^bc^	58 ± 10 ^c^
15% Amaranth-Incorporated Bread	63.3	6.65 ± 0.33 ^b^	2.37	5.53 ± 0.77 ^de^	20.95 ± 0.1 ^c^	19.27	6.57 ± 1.05 ^c^	692 ± 68 ^de^	34 ± 1.5 ^d^
30% Amaranth-Incorporated Bread	66.5	7.28 ± 0.19 ^a^	3.13	7.73 ± 2.31 ^c^	23.86 ± 0.4 ^bc^	21.95	7.53 ± 0.40 ^ab^	828 ± 110 ^cd^	45 ± 0.5 ^cd^
Whole Wheat Bread	68							1106 ± 90 ^b^	223 ± 22 ^a^

Data are analyzed using one-way ANOVA followed by Tukey’s multiple comparison test. Different letters indicate statistically significant differences between the groups (*p* < 0.05). DW: dry weight; GAE: gallic acid equivalents; QUE: quercetin equivalents.

**Table 4:** Released NH_2_ per mg protein based on total protein and free amino acid analysis and comparative digestibility assessment.

	FAA (g Leu/100 g DW)	Total Protein (g/100 g DW)	NH_2_ Released (µmol NH_2_ eq/mg Protein)	Protein Degree of Hydrolysis (%)
Germinated Quinoa	4.99	21.25	1.79 ^d^	22.1
Germinated Buckwheat	4.20	33.38	0.96 ^d^	11.9
Germinated Amaranth	3.87	30.34	0.97 ^d^	12.0
15% Quinoa-Incorporated Bread	7.50	12.10	4.73 ^ab^	58.4
30% Quinoa-Incorporated Bread	8.03	20.82	2.94 ^c^	36.3
15% Buckwheat-Incorporated Bread	7.13	8.85	6.14 ^a^	75.8
30% Buckwheat-Incorporated Bread	7.30	13.75	4.05 ^b^	50.0
15% Amaranth-Incorporated Bread	6.65	20.95	2.42 ^c^	29.9
30% Amaranth-Incorporated Bread	7.53	23.86	2.41 ^c^	29.8

Data are analyzed using one-way ANOVA followed by Tukey’s multiple comparison test. Different letters denote statistically significant differences between the groups (*p* < 0.001). DW: dry weight; FAA: free amino acid.

**Table 5:** Bioactive compound incorporation efficiency of incorporated bread compared with the whole wheat bread control.

	Bioactive Compound Content	Expected Incorporation Contribution	Actual Incorporation-Derived Content	Recovery (%)
Total phenolic compound content (mg GAE/100 g DW)				
15% Quinoa-Incorporated Bread	904 ± 56	153.2	-	-
30% Quinoa-Incorporated Bread	1009 ± 56	306.3	232	75.7
15% Buckwheat-Incorporated Bread	613 ± 45	296	-	-
30% Buckwheat-Incorporated Bread	963 ± 86	591	186	31.4
15% Amaranth-Incorporated Bread	692 ± 68	106.6	-	-
30% Amaranth-Incorporated Bread	828 ± 110	213	51	24
Total flavonoid content (mg QUE/100 g DW)				
15% Quinoa-Incorporated Bread	88 ± 1.15	53.3	-	-
30% Quinoa-Incorporated Bread	137 ± 0	107	20	19
15% Buckwheat-Incorporated Bread	38 ± 1.5	14.4	-	-
30% Buckwheat-Incorporated Bread	58 ± 10	29	-	-
15% Amaranth-Incorporated Bread	34 ± 1.5	3.3	-	-
30% Amaranth-Incorporated Bread	45 ± 0.5	6.5	-	-

Note: The bioactive compound content contributed by the whole wheat bread base is calculated proportionally according to the substitution level. For TPC: 942 and 777 mg/100 g DW at 15% and 30% substitution levels, respectively. For TFC: 190 and 157 mg/100 g DW at 15% and 30% substitution levels, respectively. DW: dry weight; GAE: gallic acid equivalents; QUE: quercetin equivalents.

## Data Availability

The original contributions presented in this study are included in the article/Supplementary Materials. Further inquiries can be directed to the corresponding author.

## Supplementary Materials

The following supporting information can be downloaded at: https://www.mdpi.com/article/10.3390/foods15183280/s1, Table S1: Total protein and FAA in vitro bioaccessibility data of germinated pseudocereals; Table S2: In vitro bioaccessibility data of total bioactive components of germinated pseudocereals.

## Abbreviations

The following abbreviations are used in this manuscript:

FAA	free amino acid
OPA	o-phthaldialdehyde
TPC	total phenolic content
TFC	total flavonoid content
PG	post-gastric
IN	intestinal
