# Supplementary material for: From Germinated Pseudocereals to Functionalized Bread: The Role of the Food Matrix in Protein and Phenolic Bioaccessibility
Source: Foods. 2026 Sep 17;15(18):3280. doi: 10.3390/foods15183280 (PMC13606043; doi:10.3390/foods15183280)
Supplement: Supplementary file 1 [file foods-15-03280-s001.zip › foods-4531458-supplementary.pdf]

Table S1. Total protein and free amino acid *in vitro* bioaccessibility data of germinated pseudocereals

	Non-digested (ND)		Postgastric (PG)	Absorbed (IN)	BAcb (%)
Total protein content (g BSA / 100 g DW)					
Germinated Quinoa	21.25±0.1[c]			No data was obtained.	
Germinated Buckwheat	33.38±0.1[a]				
Germinated Amaranth	30.34±0.78[b]				
Free amino acid / Total amino acid content (g LEU / 100 g DW)					
	FAA	*TAA*			
Germinated Quinoa	4.99±0.02[c]	19.55	5.83±0.06[b]	12.03±0.32[a]	61.5
Germinated Buckwheat	4.2±0[c]	30.70	8.27±0.15[b]	10.37±0.47[a]	33.7
Germinated Amaranth	3.87±0.06[c]	27.9	4.80±0.10[b]	8.57±0.21[a]	30.7

In vitro simulated free amino acid data were evaluated by one-way ANOVA, ignoring the dependency of the data, and all data were found to be significant with $p < 0.001$.

Table S2. *In vitro* bioaccessibility data of total bioactive components of germinated pseudocereals

	Non-digested (ND)	Postgastric (PG)	Absorbed (IN)	BAcb (%)
Total phenolic content (mg GAE / 100 g DW)				
Germinated Quinoa	1030±17[b]	733±0[b]	537±9[c]	52
Germinated Buckwheat	1988±69[a]	1524±95[a]	802±11[b]	40
Germinated Amaranth	717±49[d]	307±0[c]	257±17[c]	35.8
Total flavonoid content (mg QUE / 100 g DW)				
Germinated Quinoa	359±3[a]	106±35[b]	157±23[b]	43.7
Germinated Buckwheat	97±1[b]	66±42[b]	90±44[b]	92.7
Germinated Amaranth	22±0[c]	-	5±0[c]	22.7

Data were analyzed using one-way ANOVA followed by Tukey multiple comparison tests. Different letter grades indicate a statistically significant difference between groups ($p<0.05$).
